# Housing conditions affect enterocyte death mode and turnover rate in mouse small intestine

**DOI:** 10.1038/s41598-023-47660-1

**Published:** 2023-11-22

**Authors:** Yosuke Matsuoka, Yoshihide Tsujimoto

**Affiliations:** 1 Department of Oncogenesis and Growth Regulation, Osaka International Cancer Institute, 3-1-69 Otemae, Chuo-ku, Osaka, 541-8567 Japan; 2 Department of Molecular and Cellular Biology, Osaka International Cancer Institute, 3-1-69 Otemae, Chuo-ku, Osaka, 541-8567 Japan

**Keywords:** Cell biology, Gastroenterology

## Abstract

Small intestinal enterocytes are continuously renewed. Shedding/death of enterocytes involves receptor-interacting protein kinase 1 (RIPK1)-dependent (but RIPK3-independent) necrotic death, but the regulatory mechanism of the processes is not fully understood. Here, we show that mouse housing conditions, such as the type of bedding material and the presence or absence of a Shepherd Shack, affect enterocyte turnover rate and determine whether enterocyte shedding/death is RIPK1-independent or -dependent. Mice housed with ALPHA-dri (αDri, hard paper chip) bedding material without a Shepherd Shack had a higher, largely RIPK1-dependent enterocyte turnover rate and higher blood corticosterone levels, suggesting the involvement of minor stress, whereas mice housed with αDri plus a Shepherd Shack or with Soft Chip had a lower, RIPK1-independent turnover rate and lower blood corticosterone levels. Corticosterone administration to a small intestine culture derived from mice housed with αDri plus a Shepherd Shack or with Soft Chip increased enterocyte shedding/death and turnover. By using kinase inhibitors and knockout mice, we showed that the switch from RIPK1-independent to RIPK1-dependent enterocyte shedding/death and turnover involves suppression of TANK-binding kinase 1. Our results demonstrate that housing conditions may cause minor stress, which alters the mode of enterocyte shedding/death and enterocyte turnover rate in mice.

## Introduction

Small intestinal enterocytes are continuously renewed. These cells arise in the crypts and then migrate along the crypt-villus axis while differentiating. The enterocytes then migrate further to the tips of villi and undergo shedding into the intestinal lumen. Receptor-interacting protein kinase 1 (RIPK1) plays a role in maintaining intestinal homeostasis by regulating cell death and mediating inflammation^1–7^. We previously reported that enterocyte shedding (which involves a cell death mechanism) in normal, wild-type (WT) mouse small intestine takes place mainly by non-apoptotic cell death, which depends on RIPK1 and is sensitive to the RIPK1 kinase inhibitor necrostatin-1 (Nec-1). However, this form of non-apoptotic cell death is not dependent on RIPK3^1^, indicating that enterocyte death does not occur by necroptosis, but by a novel RIPK1-dependent death mechanism. In contrast, some groups reported that RIPK1 protects epithelial cells in mouse small intestine in a kinase-independent manner because enterocyte-specific RIPK1 knockout, but not RIPK1 kinase-dead knock-in, caused enterocyte apoptosis and premature death in mice^2,3^.

In this study, we show that mouse housing conditions affect enterocyte turnover rate and determine the RIPK1-dependence of enterocyte shedding/death. The differences in housing conditions are associated with differences in psychological stress as assessed by blood corticosterone levels. The switch from RIPK1-independent to RIPK1-dependent shedding/death and turnover involves suppression of corticosterone-sensitive TANK-binding kinase 1 (TBK1). Our results demonstrate that housing conditions may cause minor stress, which alters the mode of enterocyte shedding/death and enterocyte turnover rate in mice. This is the first to show that the mechanism of programmed cell death involved in a single phenomenon (in this study, enterocyte shedding) changes depending on the housing condition of mice.

## Results

### Bedding material-dependent change in enterocyte turnover rate and mode of shedding/death

**Figure 1:**
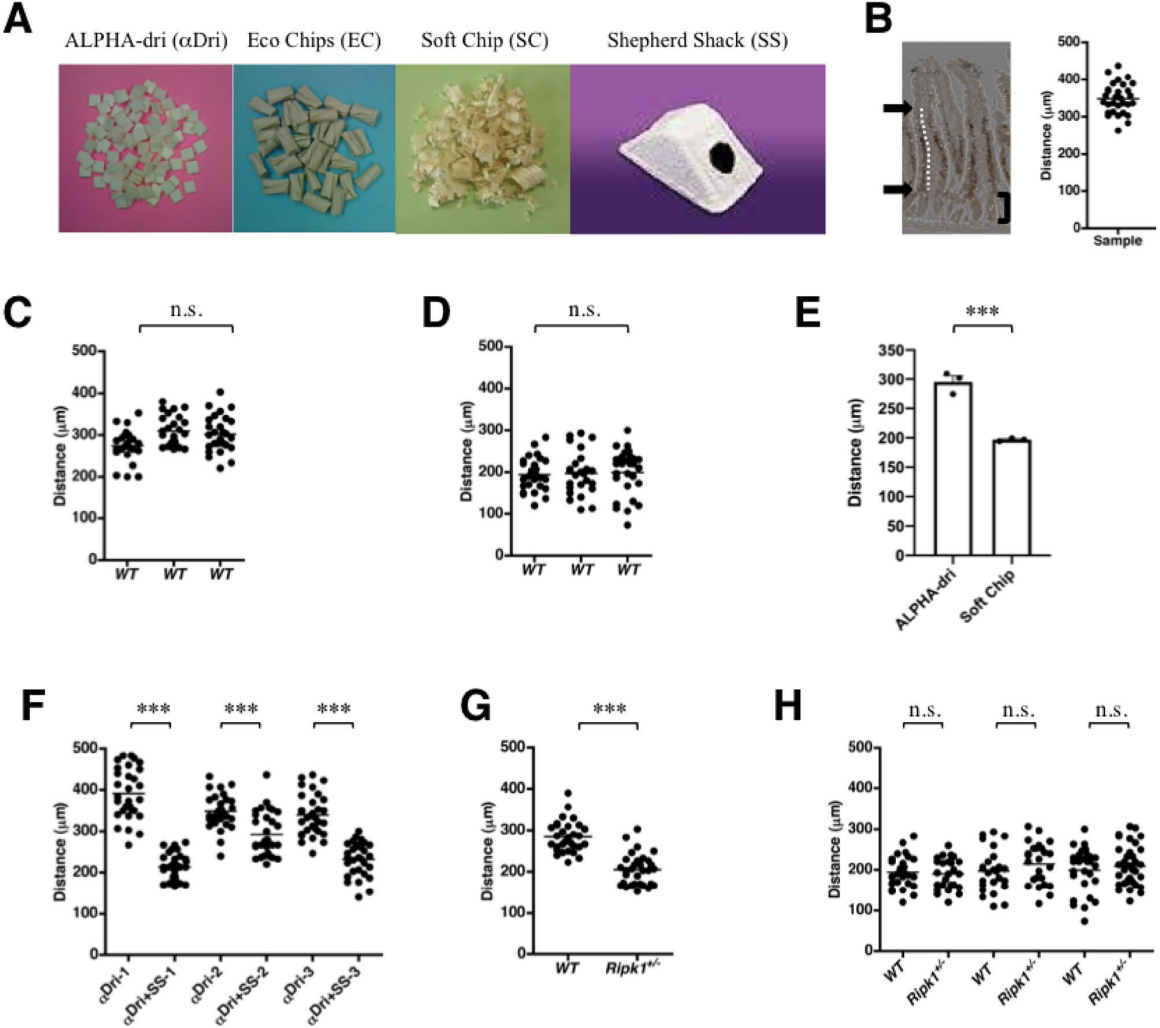
Housing conditions affect enterocyte turnover and shedding. (**A**) Housing materials used in this study. The image of a Shepherd Shack is from the website of EP Trading Co. Ltd. (Tokyo, Japan). (**B**) Evaluation of turnover rate. The distance from the villus-crypt junction to the leading BrdU-labeled cell in each villus of the duodenum (distance between arrows, indicated by a white dotted line; hereafter designated as *Distance*) was measured and plotted. More than 25 plots (usually around 30 plots) are shown; a bracket indicates a crypt region, and the horizontal line represents the mean. In each turnover rate comparing experiment, we did not notice the difference of villous length between the groups. (**C**) Enterocyte turnover rate in WT mice housed with αDri (n = 3). *Distance* was plotted; the horizontal line represents the mean. (**D**) Enterocyte turnover rate in WT mice housed with Soft Chip (n = 3). *Distance* was plotted; the horizontal line represents the mean. (**E**) The mean value in αDri (data from **C**) was compared with that in Soft Chip (data from **D**). Data are the mean ± SEM from three independent experiments. (**F**) Enterocyte turnover rate in WT mice housed with αDri was higher than that in sex-matched littermate WT mice housed with αDri + Shepherd Shack. Three pairs of mice were used. *Distance* was plotted; the horizontal line represents the mean. (**G**) When mice were housed with αDri, enterocyte turnover rate was higher in WT than in *Ripk1*^+*/−*^ mice. A pair comprising a WT and a sex-matched littermate *Ripk1*^+*/−*^ mouse was used. *Distance* was plotted; the horizontal line represents the mean. (**H**) When mice were housed with Soft Chip, enterocyte turnover rate was not different between WT and *Ripk1*^+*/−*^ mice. Three pairs comprising a WT and a sex-matched littermate *Ripk1*^+*/−*^ mouse were used. The data of the WT mice are the same as those shown in (**D**). *Distance* was plotted; the horizontal line represents the mean. For (**C**–**H**), *P* values were calculated using two-tailed unpaired *t*-tests. ***P < 0.001, n.s., not significant.

We recently noticed that when mice were housed with different bedding materials (Fig. 1A), the turnover rate of small intestinal enterocytes was significantly different. The turnover rate was assessed by measuring the migration distance of the leading BrdU-labeled enterocytes, as described (Fig. 1B). Enterocyte turnover rate in WT mice housed with soft wood chips “Soft Chip” was about two-thirds lower than that in WT mice housed with hard paper chips “ALPHA-dri” (αDri), which was used in our previously reported studies^1^ (Fig. 1C–E). Therefore, we decided to investigate how different bedding materials, such as Soft Chip and αDri, affect enterocyte shedding/death and turnover. Because mice can hide themselves in Soft Chip but not in αDri, we hypothesized that the presence or absence of mild psychological stress might cause the differences in enterocyte turnover rate. To test this possibility, we placed a paper shelter “Shepherd Shack”, in which the mice could hide themselves, in mouse cages with αDri. In this housing condition, enterocyte turnover rate was reduced to the same level as that observed with Soft Chip (Fig. 1F). More importantly, WT and *Ripk1*^+*/−*^ mice had different turnover rates of enterocytes when housed with αDri (Fig. 1G), as we have previously reported^1^, but no difference was observed between WT and *Ripk1*^+*/−*^ mice housed with Soft Chip (Fig. 1H), indicating that enterocyte turnover is independent of RIPK1 in mice housed with Soft Chip but dependent on RIPK1 in mice housed with αDri. Note that since *Ripk1*^-/-^ mice are embryonic lethal, we utilized RIPK1 heteroinsufficiency to validate the RIPK1-dependence of enterocyte turnover as previously reported^1^.

**Figure 2:**
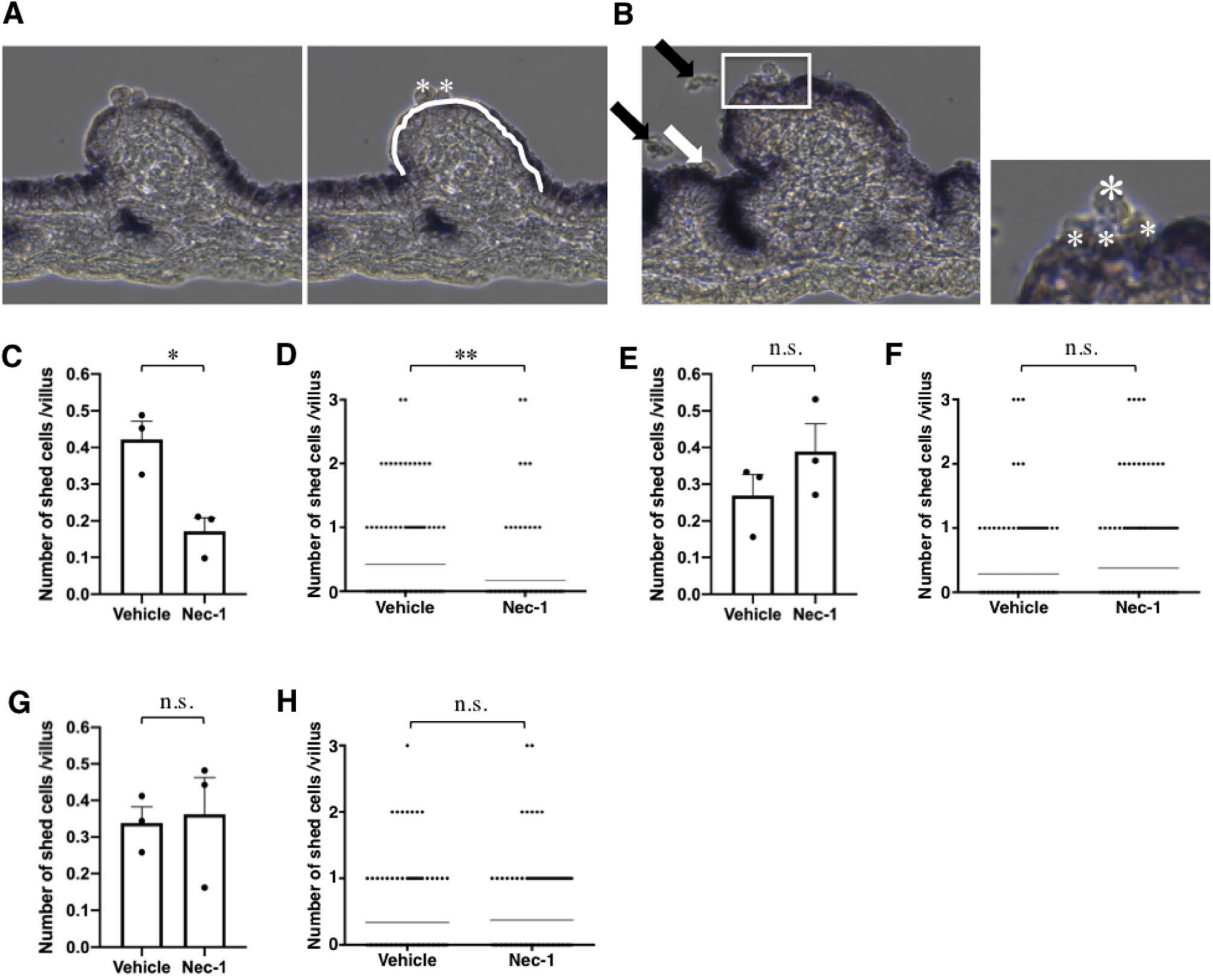
Housing conditions affect the mode of enterocyte shedding/death. (**A**, **B**) Evaluation of enterocyte shedding using regenerated villi (RV) cultures. To evaluate enterocyte shedding, shed cells (indicated by * in **A**) from each villus (indicated by a white line) were counted. Cells fully detached from the villus (black arrows in **B**) and those in the inter-villous region (white arrow in **B**) were NOT counted. In (**B**), the area surrounded by a white rectangle is shown enlarged in the inset to the right. Shed cells directly associated with a villus (indicated by a smaller * in the inset in **B**) were counted, but those indirectly associated (indicated by a larger * in the inset in B) were NOT counted, because of aiming to count only newly shed cells. More than 30 villi were counted. (**C**, **D**) Nec-1 inhibited enterocyte shedding in RV culture derived from mice housed with αDri. (**C**) Counts of shed cells; data are the mean ± SEM from three independent experiments. In (**D**), all plots of the three experiments are shown (vehicle: n = 128, Nec-1: n = 118); the horizontal line represents the mean. (**E**, **F**) Nec-1 did not inhibit enterocyte shedding in RV culture derived from mice housed with αDri + Shepherd Shack. (**E**) Counts of shed cells; data are the mean ± SEM from three independent experiments. In (**F**), all plots of the three experiments are shown (vehicle: n = 152, Nec-1: n = 185); the horizontal line represents the mean. (**G**, **H**) Nec-1 did not inhibit enterocyte shedding in RV culture derived from mice housed with Soft Chip. (**G**), Counts of shed cells; data are the mean ± SEM from three independent experiments. In (**H**), all plots of the three experiments are shown (vehicle: n = 118, Nec-1: n = 134); the horizontal line represents the mean. For (**C**, **E**, **G**), *P* values were calculated using two-tailed unpaired *t*-tests. For (**D**, **F**, **H**), *P* values were calculated using Mann–Whitney *U*-tests. *P < 0.05, **P < 0.01, n.s., not significant.

We previously showed that enterocyte shedding/death and turnover also occur in the small intestine culture system we developed (which we named regenerated villi [RV] culture)^1^. In this system, we measured the number of shed cells as described (Fig. 2A,B). In addition to evaluating cell shedding/death, this system also allows us to examine cell proliferation and migration separately, and we found that Nec-1 inhibited cell shedding/death but not proliferation or migration^1^. Therefore, we concluded that the reduced turnover rate of enterocytes observed in *Ripk1*^+*/−*^ mice is due to the partial suppression of enterocyte shedding/death in these mice with haploinsufficiency of RIPK1^1^. Although enterocyte shedding in RV culture derived from mice housed with αDri was Nec-1 sensitive (Fig. 2C,D) as previously described^1^, this was not the case in RV culture derived from mice housed with αDri plus a Shepherd Shack (Fig. 2E,F); these findings are consistent with the in vivo results obtained in *Ripk1*^+*/−*^ mice. Similar to the difference in turnover rate observed in vivo, the number of shed cells was higher in RV culture derived from mice housed with αDri than in RV culture derived from mice housed with αDri plus a Shepherd Shack (Fig. 2C,E). We also observed that enterocyte shedding/death in RV culture derived from mice housed with Soft Chip was not sensitive to Nec-1 (Fig. 2G,H). The results obtained in Soft Chip were reproduced in mice housed with Eco Chips (see *Ripk3*^*−/−*^ data in Fig. 5B,C). These findings indicate that the housing condition-dependent differences in the mode of enterocyte shedding/death and turnover rate are also found in RV culture. Taken together, the data indicate that bedding materials affect enterocyte shedding/death and turnover rate.

### The stress hormone corticosterone alters the mode of enterocyte shedding/death and increases turnover rate

**Figure 3:**
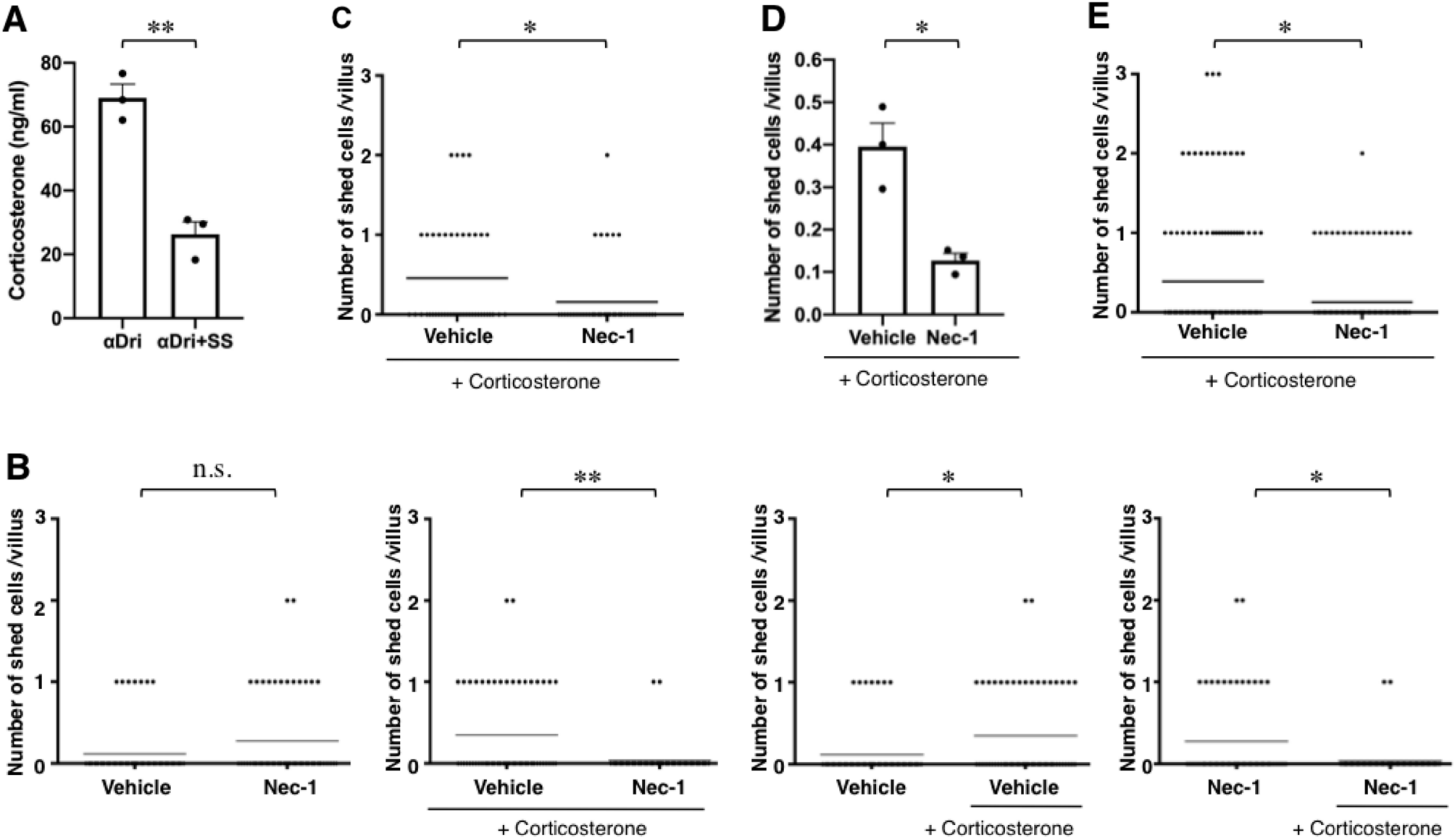
Corticosterone changes the mode of cell death in RV culture. (**A**) Plasma corticosterone concentration at Zeitgeber time 0 in WT mice housed with αDri (n = 3) compared with that of sex-matched littermate WT mice housed with αDri + Shepherd Shack (n = 3). (**B**, **C**) Nec-1 inhibits enterocyte shedding in corticosterone-treated RV culture derived from mice housed with αDri + Shepherd Shack. (**B**) Villi regenerated for 21 h in the absence or presence of 50 ng/mL of corticosterone were treated with vehicle alone or 20 μM Nec-1 for 3 h. The figures show a representative plot of the number of shed cells in RV culture treated with vehicle (n = 61), and Nec-1 (n = 59), corticosterone (50 ng/mL)/vehicle (n = 61), and corticosterone (50 ng/mL)/Nec-1 (n = 56) (left two panels). For comparison, a data set of vehicle without or with corticosterone and that of Nec-1 without or with corticosterone are shown (right two panels). In (**C**), another data set of RV culture treated with corticosterone (50 ng/mL)/vehicle (n = 44) and corticosterone (50 ng/mL)/Nec-1 (n = 45) is shown. (**D**, **E**) Nec-1 inhibits enterocyte shedding in corticosterone-treated RV culture derived from mice housed with Soft Chip. (**D**), Villi regenerated for 21 h in the absence or presence of 100 ng/mL of corticosterone were treated with vehicle alone or 20 μM Nec-1 for 3 h. Cells shed in these experiments were counted; data are the mean ± SEM from three independent experiments. In (**E**), all plots of the three experiments are shown (vehicle: n = 149, Nec-1: n = 150); the horizontal line represents the mean. For (**A**, **D**), *P* values were calculated using two-tailed unpaired *t*-tests. For (**B**, **C**, **E**), *P* values were calculated using Mann–Whitney *U*-tests. *P < 0.05, **P < 0.01, n.s., not significant.

To examine whether the differences in housing conditions described above were associated with differences in psychological stress, we measured blood levels of the stress hormone corticosterone. The blood level of corticosterone is known to show a circadian rhythm and to be lowest in mice at Zeitgeber time 0 (ZT0)^8^. We found that at ZT0, the corticosterone level was higher in mice housed with αDri than in mice housed with αDri with a Shepherd Shack (Fig. 3A) or in Soft Chip or Eco Chips (Fig. S1). As described above, enterocyte shedding in RV culture derived from mice housed with αDri was Nec-1 sensitive, whereas that derived from mice housed with αDri with a Shepherd Shack or in Soft Chip or Eco Chips was not. To examine the effects of corticosterone on enterocyte shedding, we added corticosterone to RV cultures derived from mice housed with αDri with a Shepherd Shack. The results showed that corticosterone stimulated enterocyte shedding and that this stimulation was sensitive to Nec-1, i.e., RIPK1-dependent (Fig. 3B,C). We obtained the same results when we used Soft Chip (Fig. 3D,E) or Eco Chips (data not shown). Thus, the effects of different housing conditions on enterocyte shedding described above can be replicated by performing RV culture with or without corticosterone, indicating that corticosterone plays a crucial role in the enhancement of enterocyte shedding/death and turnover rate.

### TBK1 suppresses RIPK1-dependent death

**Figure 4:**
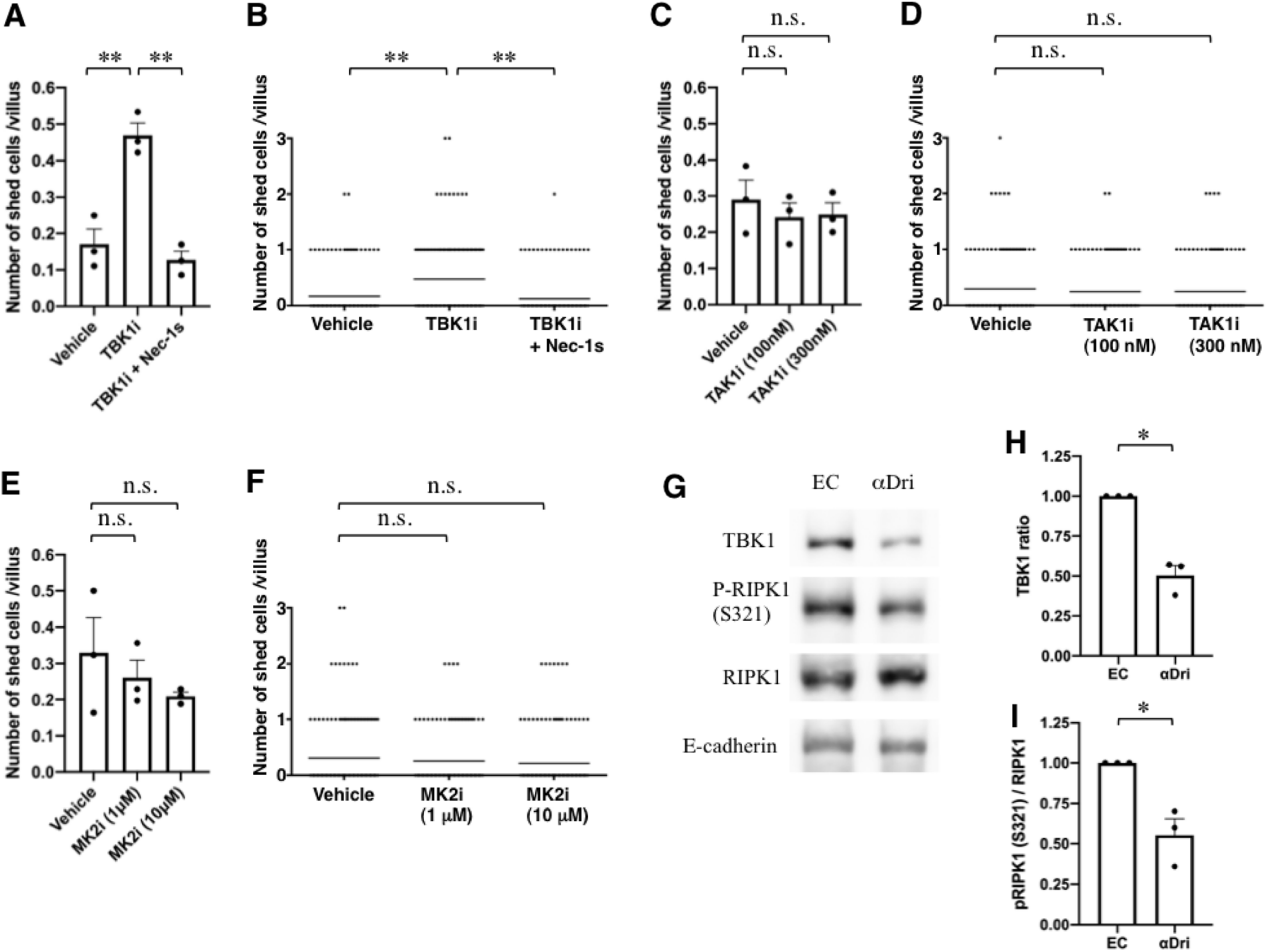
Inhibition of TBK1 results in stimulation of RIPK1-dependent enterocyte shedding. (**A**–**F**) TBK1i stimulated enterocyte shedding in RV culture, but TAK1i and MK2i did not. RV were treated with 2 µM TBK1i (MRT67307) (**A**, **B**), 100 or 300 nM TAK1i (5Z-7-Oxozeaenol) (**C**, **D**), or 1 or 10 µM MK2i (PF-3644022) (**E**, **F**) for 6 h. In the case of TBK1i, RV were also pre-treated for 30 min with Nec-1s, and then 2 µM TBK1i was added for 6 h. Shed cells were counted; data are the mean ± SEM from three independent experiments (**A**, **C**, **E**). In (**B**, **D**, **F**), all plots of the three experiments are shown (**B**: vehicle, n = 159, TBK1i, n = 163, TBK1i + Nec-1s, n = 151, **D**: vehicle, n = 142 , TAK1i [100 nM], n = 127, TAK1i [300 nM], n = 130, **F**: vehicle, n = 181, MK2i [1 µM], n = 151, MK2i [10 µM], n = 167); the horizontal line represents the mean. (**G**–**I**) The amount of TBK1 and the S321-phosphorylation level of RIPK1 were lower in WT mice housed with αDri than in sex-matched littermate WT mice housed with Eco Chips. (**G**), Western blot analysis of the quantity of TBK1, S321-phosphorylated-RIPK1 (p-RIPK1 [S321]), and total RIPK1 (RIPK1) in the enterocytes of WT mice housed with αDri and sex-matched littermate WT mice housed with Eco Chips. E-cadherin was a loading control. (**H**), Densitometry of TBK1 was normalized to E-cadherin; then, the ratio (relative to a value of Eco Chips as 1) was calculated in each Eco Chips/ αDri pair. The mean ± SEM from three pairs is shown. (**I**), Densitometry was performed to obtain the ratio of p-RIPK1 (S321) to total RIPK1 (normalized to Eco Chips as 1) in each Eco Chips/αDri pair. The mean ± SEM from three pairs is shown. For (**A**–**F**),* P* values were calculated using one-way Anova with Dunnett’s multiple comparison tests. For (**H**, **I**), *P* values were calculated using one-sample *t*-tests. *P < 0.05, **P < 0.01, n.s., not significant. Uncropped Western blot data used in **G**, **H**, and **I** are shown in supplementary Fig. S7.

TBK1^9,10^, transforming growth factor β-activated kinase 1 (TAK1)^11,12^, and mitogen-activated protein kinase-activated protein kinase 2 (MK2)^13^ were recently reported to suppress RIPK1-mediated cell death. Interestingly, these kinases are glucocorticoid sensitive^14–16^, raising the possibility that they may be involved in the effects of different housing conditions on enterocyte shedding/death and turnover. To examine this possibility, we treated RV culture derived from mice housed with Eco Chips with the TBK1 inhibitor (TBK1i) MRT67307, the TAK1 inhibitor (TAK1i) 5Z-7-Oxozeaenol, and the MK2 inhibitor (MK2i) PF-3644022. The TBK1i (Fig. 4A,B) stimulated enterocyte shedding/death, and this stimulation was sensitive to necrostatin-1s (Nec-1s, a more specific RIPK1 inhibitor than Nec-1), whereas the TAK1i (Fig. 4C,D) and MK2i (Fig. 4E,F) did not enhance enterocyte shedding/death. These results suggest an important role of TBK1 in the housing condition-dependent change in enterocyte shedding/death and turnover rate.

Next, we examined whether housing condition affects the amount of TBK1. We found that the amount of TBK1 in villous epithelial cell extracts was higher in mice housed with Eco Chips than in mice housed with αDri (Fig. 4G,H). Toward understanding underlying mechanisms for change in TBK1 level with different housing conditions, we tried to utilize organoid cultures. Reduction in TBK1 in villous epithelial cells of mice housed with αDri was shown using isolated enterocytes depleted from other types of cells in small intestine. Since enterocytes could not be isolated from RV cultures, we utilized organoids which consist mainly of enterocytes. Using small intestinal organoids (Fig. S2), we found that the amount of TBK1 decreased when organoids were treated with corticosterone (Fig. S3A,B) and that mRNA level of TBK1 was only slightly affected by corticosterone (Fig. S3C), suggesting the reduction of TBK1 by corticosterone was mainly dependent on post-transcriptional events. Phosphorylation of serine321 (S321) of RIPK1 was reported to inhibit RIPK1 activation^13^, and this inhibition was thought to be carried out by MK2, TAK1, TBK1, and inhibitor of nuclear factor kappa-B kinase subunit epsilon (IKKε)^9,10,13,17^. We found that mice housed with αDri had a lower S321 phosphorylation level of RIPK1 than mice housed with Eco Chips (Fig. 4G,I). These data indicate that the amount of TBK1 is related to determining the enterocyte death mode. Taken together, these results suggest that TBK1 plays a role in suppressing RIPK1-dependent enterocyte shedding/death via S321 phosphorylation of RIPK1 and that this suppression is responsible for the activation of RIPK1-dependent enterocyte shedding/death.

**Figure 5:**
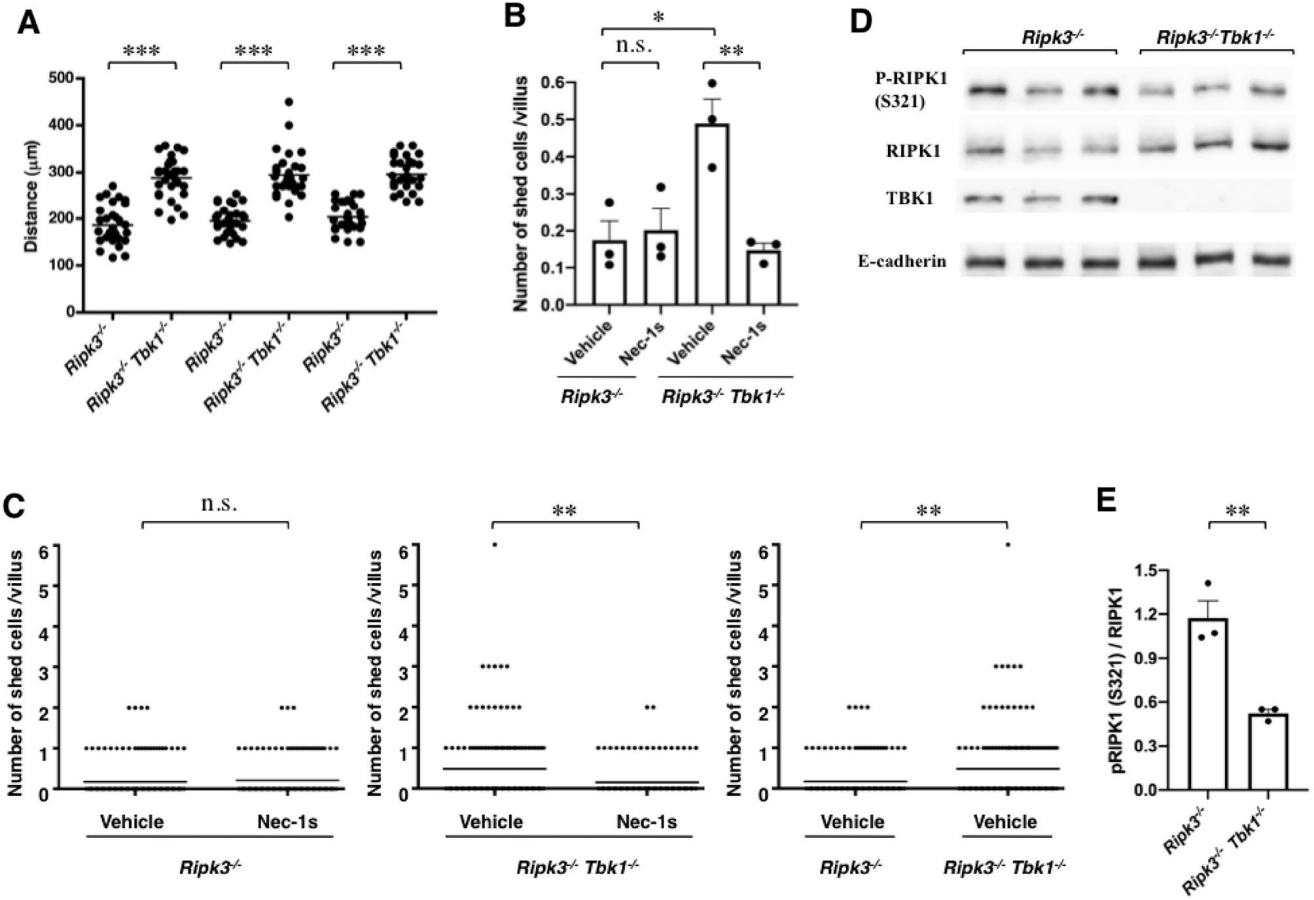
Enterocyte turnover and RIPK1-dependent enterocyte shedding were stimulated in TBK1-deficient mice. (**A**) The enterocyte turnover rates were higher in *Ripk3*^*−/−*^* Tbk1*^*−/−*^ mice than in sex-matched littermate *Ripk3*^*−/−*^ mice in three independent experiments. *Distance* was plotted; the horizontal line represents the mean. (**B**, **C**) Enterocyte shedding was stimulated in RV culture from *Ripk3*^*−/−*^* Tbk1*^*−/−*^ mice, and this stimulation was Nec-1s-sensitive. (**B**), Shed cells were counted; data are the mean ± SEM from three independent experiments. In (**C**), all plots of the three experiments are shown (*Ripk3*^*−/−*^: vehicle, n = 203, Nec-1s, n = 172, *Ripk3*^*−/−*^* Tbk1*^*−/−*^: vehicle, n = 165, Nec-1s, n = 141); the horizontal line represents the mean. For comparison, vehicle and Nec-1s were paired in each genotype and vehicles of each genotype were also paired. (**D**, **E**) Western blot showing the levels of S321-phosphorylated-RIPK1 (p-RIPK1 [S321]), total RIPK1 [RIPK1], and TBK1 in enterocytes of *Ripk3*^*−/−*^ (n = 3) and *Ripk3*^*−/−*^* Tbk1*^*−/−*^ mice (n = 3) (**D**). E-cadherin is a loading control. (**E**), Densitometry was performed to obtain the ratio of p-RIPK1 (S321) to total RIPK1. The mean ± SEM from three mice is shown. For (**A**, **E**), *P* values were calculated using two-tailed unpaired *t*-tests. For (**B**), *P* values were calculated using two-way ANOVA with Bonferroni post-tests. For (**C**), *P* values were calculated using Mann–Whitney *U*-tests. *P < 0.05, **P < 0.01, ***P < 0.001, n.s., not significant. Uncropped Western blot data used in D are shown in supplementary Fig. S8.

To confirm the inhibitory effect of TBK1 on RIPK1-dependent enterocyte shedding/death, we used *Tbk1*^*−/−*^ mice. However, *Tbk1*^*−/−*^ mice die as embryos^18^, so we tried to obtain *Ripk3*^*−/−*^* Tbk1*^*−/−*^ mice because about one-third of these types of mice reportedly survive to adulthood^9^. Our previous study with the αDri housing condition showed that the enterocyte turnover rate in *Ripk3*^*−/−*^ mice was not different from that in WT mice and that Nec-1 inhibits enterocyte shedding in RV culture from *Ripk3*^*−/−*^ mice^1^. Here, we found that in mice housed with Eco Chips, the enterocyte turnover rate was higher in *Ripk3*^*−/−*^*Tbk1*^*−/−*^ mice than in *Ripk3*^*−/−*^ mice (Fig. 5A). Mucosa enterocyte shedding was also higher in RV-cultured *Ripk3*^*−/−*^*Tbk1*^*−/−*^ than in RV-cultured *Ripk3*^*−/−*^ mucosa, and shedding was Nec-1s sensitive (Fig. 5B,C). We confirmed that the blood level of corticosterone was almost the same in *Ripk3*^*−/−*^*Tbk1*^*−/−*^ mice as in WT mice (Fig. S1). In addition, we examined whether the differences in the enterocyte turnover rate between mice housed with αDri and those housed with Eco Chips were no longer found in *Ripk3*^*−/−*^*Tbk1*^*−/−*^ mice. As a result, no differences in turnover rate were observed (Fig. S4), indicating that TBK1 plays an important role in suppressing RIPK1 to inhibit RIPK1-dependent enterocyte shedding/death and turnover.

The activation and activity of RIPK1 are regulated by various post-translational modifications, such as phosphorylation and ubiquitination^19^. S166 is known to be autophosphorylated when RIPK1 is active^17^. However, we did not detect S166 phosphorylation of RIPK1 by immunoblotting analysis of enterocytes (Fig. S5), which might be because enterocyte shedding occurs only in a limited region and RIPK1 activation is likely to occur in only a very small population of cells. We compared the S321 phosphorylation level of RIPK1 in *Ripk3*^*−/−*^*Tbk1*^*−/−*^ and *Ripk3*^*−/−*^ enterocytes and found that it was lower in *Ripk3*^*−/−*^*Tbk1*^*−/−*^ mice (Fig. 5D,E). As described above, the S321 phosphorylation level of RIPK1 in mice housed with αDri was lower than that in mice housed with Eco Chips (Fig. 4G,I). Moreover, when small intestinal organoids were treated with TBKi, we found that the S321 phosphorylation level decreased (Fig. S6). These data suggest that changing the S321 phosphorylation level might be one of the mechanisms by which TBK1 suppresses RIPK1 in enterocytes.

## Discussion

We demonstrated that housing conditions affect turnover rate and mode of enterocyte shedding/death in mouse small intestine. When hard paper chip αDri bedding material alone is used, the turnover rate of enterocytes increases and RIPK1-dependent enterocyte shedding/death dominates, whereas when αDri is combined with a Shepherd Shack or when Soft Chip or Eco Chips are used, RIPK1-independent enterocyte shedding/death dominates and the turnover rate is lower. Mice housed with αDri have higher blood levels of corticosterone, and administration of corticosterone to organ cultures (RV cultures) derived from mice housed with αDri + Shepherd Shack or with Soft Chip increases enterocyte turnover/shedding, which becomes RIPK1-dependent. These results strongly suggest that in mice, housing condition-related psychological stress changes the cell death mode of enterocytes from RIPK1-independent to RIPK1-dependent and accelerates enterocyte turnover. Interestingly, the differences in enterocyte turnover rate and in the RIPK1-dependency of enterocyte shedding/death caused by different housing conditions were well replicated in RV organ cultures, indicating that the mechanisms controlling the effects of housing conditions on enterocyte shedding/death and turnover processes are maintained after dissecting the intestine out from mice and culturing it in vitro for at least two days. Recently, a study reported that the gut microbiome regulates psychological stress-induced inflammation^20^. However, because the gut microbiome was not maintained in our organ culture system, our data indicate that the microbiome may not play a role in the effects of housing conditions. We hypothesize that the in vivo effects of the stress hormone corticosterone in mice might be maintained in cultures for a while and thus maintain the enhanced enterocyte shedding/death and turnover. It remains to be determined whether the main mechanisms of RIPK1-dependent and RIPK1-independent enterocyte shedding/death are completely different or share some common steps.

RIPK1 is regulated by different means, such as phosphorylation and ubiquitination^19^. Several upstream kinases of RIPK1, including TBK1^9,10^, TAK1^11,12^, and MK2^13^, are suppressed by the hormone glucocorticoid^14–16^. The experiments using kinase inhibitors and knockout mice showed that TBK1 plays an important role in suppressing RIPK1 in enterocytes. Although the precise mechanisms by which TBK1 suppresses RIPK1 in enterocytes and minor stress inhibits TBK1 (resulting in activation of RIPK1) need to be determined, we suggest that TBK1-dependent phosphorylation of S321, which is known to suppress RIPK1 activity, is one of the mechanisms by which TBK1 suppresses RIPK1 and that stress suppresses the expression level of TBK1 in enterocytes.

Acceleration of enterocyte turnover is primarily thought to be a strategy to eliminate harmful factors from epithelia. Interestingly, the enterocyte turnover rate was about 1.5-fold higher with αDri than with the other housing conditions tested in this study, and this increase is the same as that observed in mice in which a virus infection state is mimicked^21^. When human beings are affected by psychological stress, they often tend to become more susceptible to harmful pathogens, partly because stress is associated with immune system suppression. In healthy people exposed to pathogens, psychological acceleration of enterocyte turnover works as a defense system by increasing the efficiency of pathogen elimination from the epithelia. On the other hand, as reported in a recent systematic review, psychological stress has adverse effects in patients with inflammatory bowel disease (IBD)^22^. If the mode of enterocyte shedding/death is altered by psychological stress, as observed in mice, acceleration of enterocyte shedding and turnover could weaken the epithelial barrier, allowing intestinal flora to cross the epithelium. This effect could cause serious problems in patients with IBD. Indeed, a genome-wide association study identified a RIPK1 regulator, A20, as a risk factor for IBD^23^. Thus, proper control of the TBK1-RIPK1 axis might be a therapeutic target to improve IBD.

In conclusion, we demonstrated that mild stress originating from different bedding materials alters the process of shedding/death mode of enterocytes in small intestine. Further investigations of the mechanism involved in the switch of shedding/death mode found in this study will give us a new field of cell death research.

## Materials and methods

### Animals

In most experiments, we used both female and male mice because so far, we have not obtained any sex-dependent data in our experiments. C57BL/6J mice (Japan SLC, Hamamatsu, Japan), *Ripk3*^*−/−*^ mice^24^, *Tbk1*^+*/−*^ mice^19^, and *Ripk1*^+*/−*^ mice^25^ were used in this study. *Tbk1*^+*/−*^ mice were crossed with *Ripk3*^*−/−*^ mice to generate *Tbk1*^+*/−*^*Ripk3*^+*/−*^ mice; then, *Tbk1*^+*/−*^*Ripk3*^+*/−*^ mice were crossed with *Tbk1*^+*/−*^*Ripk3*^+*/−*^ mice to generate *Tbk1*^+*/−*^*Ripk3*^*−/−*^ mice; and finally, *Tbk1*^+*/−*^*Ripk3*^*−/−*^ mice were crossed with *Tbk1*^+*/−*^*Ripk3*^*−/−*^ mice to generate *Tbk1*^*−/−*^*Ripk3*^*−/−*^ mice and their littermate control *Ripk3*^*−/−*^ mice. Mice aged 12–24 weeks (in some cases, 32 weeks and 13 months) fed an unrestricted diet were used in this study. Mice were maintained at 23 ± 1.5 °C, under a 12:12 light–dark cycle. All methods were performed in accordance with the relevant guidelines and regulations. The experimental protocol was approved by the Ethical Review Committee for Animal Experimentation of Osaka University Medical School, Osaka Medical Center for Cancer and Cardiovascular Diseases, and Osaka International Cancer Institute. All methods are reported in accordance with ARRIVE guidelines.

### Mouse housing conditions

To compare genotypes, we used only the pairs of sex-matched littermates reared in the same cage. If male littermates fought each other, they were separated and omitted from the experiments. When we used Eco Chips (CLEA Japan, Tokyo, Japan) as bedding material, mice were housed in Innocages (ORIENTAL, Osaka, Japan, width, 234 mm; depth, 373 mm; height, 140 mm; individually ventilated). In the case of Soft Chip (Japan SLC), cages (CLEA Japan, width, 174 mm; depth, 278 mm; height, 131 mm) with 35 g of Soft Chip were used. To examine the enterocyte turnover rate in the small intestine of mice housed with αDri (Shepherd Specialty Papers, TN, USA) and to compare the enterocyte turnover rate of mice housed with αDri with that of mice housed with αDri plus a Shepherd Shack (Shepherd Specialty Papers), we used cages (CLEA Japan, width, 143 mm; depth, 293 mm; height, 148 mm; not ventilated) with 40 g of αDri with or without a Shepherd Shack (width, 83 mm; depth, 146 mm; height, 63 mm). For the comparative experiment, we used only pairs of sex-matched littermates that were reared in the same cage. One male (or female) mouse was housed in each condition for 6 days or more (typically 7 days) before it was used for experiments. To compare the protein amounts of enterocyte extracts in mice housed with αDri with those in mice housed with Eco Chips, one male (or female) mouse was housed in an individually ventilated Innocage with 40 g of αDri, and the sex-matched littermate that was reared in the same cage was housed in an individually ventilated Innocage with 80 g of Eco Chips. We did not use more than 40 g of αDri because some mice collected it in one place and huddled into it to hide themselves. MF (ORIENTAL YEAST, Tokyo, Japan) or DC8 (CLEA Japan) was used as food. The difference in food did not affect enterocyte turnover or shedding/death.

### Reagents

Nec-1 and Nec-1s were obtained from Merck (Hessen, Germany); zVAD-fmk, from Peptide Institute (Osaka, Japan); the TBK1 inhibitor (TBK1i) MRT67307, from Selleck (TX, USA); and the TAK1 inhibitor (TAK1i) 5Z-7-Oxozeaenol and MK2 inhibitor (MK2i) PF-3644022, from Merck. All reagents were dissolved in dimethyl sulfoxide (DMSO) except TBK1i, which was dissolved in H_2_O. PhosSTOP was obtained from Merck, and Protease Inhibitor Cocktail from Nacalai Tesque (Kyoto, Japan). The following antibodies were used: rabbit anti-ApoA1 (PA5-88109; Thermo Fisher Scientific, MA, USA), mouse anti-E-cadherin (610181; BD Biosciences, NJ, USA), mouse anti-RIPK1 (610458; BD Biosciences), rabbit anti-RIPK1 (#3493; Cell Signaling Technology, MA, USA), rabbit anti-phospho-RIPK1 (#83613; S321, mouse-specific; Cell Signaling Technology), rabbit anti-phospho-RIPK1 (#31122; S166, rodent-specific; Cell Signaling Technology), rabbit anti-phospho-RIPK1 (ARG66476; S166; Arigo Biolaboratories, Hsinchu City, Taiwan), rabbit anti-TBK1 (#3504; Cell Signaling Technology), mouse anti-BrdU biotin-XX (A21301MP; Thermo Fisher Scientific), Streptavidin-HRP (P0397; Agilent, CA, USA), HRP-labeled anti-rabbit IgG (#7074; Cell Signaling Technology), and HRP-labeled anti-mouse IgG (#7076, Cell Signaling Technology).

### Organ culture (RV culture)

Organ culture of mouse intestinal mucosa was performed as described previously^1^. The basic idea for the RV culture was that we first removed villus epithelial cells from the underlying mesenchymal tissues and then allowed the epithelial cells to regenerate from the remaining crypts so that they covered the mesenchymal tissues again. Because the mesenchymal tissues shrank considerably in culture, the RV were much shorter than in vivo villi. RV cultures can be maintained for at least two days. A brief technical description is as follows: A segment of jejunum was opened longitudinally, washed in Hanks’ balanced salt solution (HBSS) without calcium and magnesium {HBSS (-)}, and incubated on ice for 60–90 min in this solution. Then, the segment was pinned flat to a polyvinyl board and flushed vigorously with HBSS (-) to remove the epithelial cells from the villi. Subsequently, the mucosa was placed on a filter with the villus side up and cut into pieces, which were placed onto a stainless mesh in a center-well organ culture dish (353037; Corning, NY, USA) for air–liquid interface culture. Medium (L-15 medium supplemented with 10% [v/v] fetal bovine serum [FBS], 2 mM glutamine, and antibiotics or Dulbecco's Modified Eagle's Medium (DMEM) supplemented with 10% [v/v] FBS and antibiotics) was added to the central well, which was surrounded by an outer well containing HBSS (-). Then, culture was performed at 37 °C in 100% air or in 90% air/10% CO_2_ (in the case of DMEM) for an appropriate period. Although regeneration was complete within 6–12 h, we used 21- or 24-h cultured mucosa for most experiments. If corticosterone treatment was being applied, it was added to the medium from the beginning to the end of the culture.

### Small intestinal organoid culture

Mouse duodenal crypts were isolated as reported^26^ with minor modifications. We used 2.5 mM EDTA instead of 2 mM EDTA. Duodenal organoids were established as reported^27^ with minor modifications. We used 48-well plate with 200–250 uL/well of organoid culture medium instead of 24-well plate with 500 uL/well of the medium. To maintain organoids, medium was changed every 2 days and organoids were passaged by mechanical dissociation with pipetting as reported^28^. We used three types of organoid culture medium, IntestiCult organoid growth medium^27^ (OGM, purchased from STEMCELL technologies, BC, Canada), ENR medium^26,28–30^, and ER medium^29^. ENR medium is a prototype of small intestinal organoid growth medium which contains EGF, noggin, and R-spondin 1. ER medium is noggin-depleted ENR medium. In OGM, organoids were kept undifferentiated, forming spheres. When they were cultured in ER medium, they differentiated, exhibiting darker structures with some buds (we change OGM to ENR for 0–2 days and then changed ENR to ER). When we treated differentiated organoids with corticosterone or TBKi, we used differentiated organoids cultured in ER medium for 3–5 days. Since ER medium contains B-27 supplement which contains corticosterone, we did not add B-27 supplement, instead added nicotinamide which was reported to improve culture condition^30^.

### In vivo experiments with BrdU

To assess enterocyte turnover rate, BrdU (100 mg/kg) was injected intraperitoneally. After 48 h, mice were euthanized, and the duodenum was removed for further analysis. After anti-BrdU staining, the enterocyte turnover rate was measured as described in Fig. 1B.

### Corticosterone measurement

Blood samples were collected from euthanized or live mice (in the latter case, within 1 min of disturbance), kept on ice, and then centrifuged at 3000 rpm for 10 min. Plasma samples were obtained and stored at − 80 °C. Corticosterone was measured with a commercially available enzyme-linked immunoassay kit (Yanaihara, Fujinomiya, Japan).

### Tissue sample preparation and immunohistochemistry

In principle, tissue sample preparation and immunohistochemistry were performed as described previously^1^. The duodenum of each mouse was sliced into 1- or 1.5-cm segments and fixed in neutral buffered formalin without washing away the luminal contents. Cultured mucosal segments were also fixed in neutral buffered formalin without washing. Then, samples were cryoprotected in 30% sucrose in phosphate-buffered serum (PBS) overnight, after which sections were cut on a cryostat. For alkaline phosphatase (AP) staining, sections were developed in AP buffer (100 mM Tris–HCl pH9.5, 100 mM NaCl, 5 mM MgCl_2_) containing 0.3 mg/ml of nitro blue tetrazolium and 0.15 mg/ml of bromochloroindolyl phosphate. For immunohistochemistry, sections were treated with 0.5% Triton X-100 in PBS, and then endogenous peroxidase activity was blocked by incubation with 0.3% hydrogen peroxide in PBS for 30 min. DNA denaturation was performed by treating sections with 4N HCl for 15 min at room temperature. After washing with PBS supplemented with 0.1% Tween 20 (PBS-T), incubation with anti-BrdU biotin-XX (1:200) was performed in blocking solution (3% bovine serum albumin [BSA] in PBS supplemented with 0.1% NaN_3_) overnight at 4 °C. After washing with PBS-T, the sections were incubated with avidin-HRP (1:400) in PBS-T for 60 min at room temperature. After a final wash with PBS-T, the sections were developed with ImmPACT DAB (Vector, CA, USA). All images were collected with a Plan Fluor 10x/0.30 objective (Nikon, Tokyo, Japan) or an S Plan Fluor 20x/0.45 ELWD objective (Nikon) on a microscope (BZ-X710; Keyence, Osaka, Japan).

### Counting the number of shed cells in RV culture

Sections stained with AP were used for counting. The number of shed cells was counted as described in Fig. 2A,B.

### Immunoblotting

Epithelial cells were detached from the mucosa and subjected to immunoblotting as described previously^1^. Small intestinal organoids (typically cultured in 2 wells, corresponding to 2 to 6 × 10^5^ cells) were recovered and collected as reported^31^ for each treatment. Total protein extracts were prepared by solubilizing epithelial cells or organoids in sodium dodecyl sulfate (SDS) sample buffer (50 mM Tris–HCl pH 6.8, 1% SDS, 10% glycerol, PhosSTOP, Protease Inhibitor Cocktail, and 50 mM dithiothreitol). Samples were subjected to SDS–polyacrylamide gel electrophoresis (PAGE) on a 5–20% gradient polyacrylamide gel and transferred to a polyvinylidene difluoride (PVDF) membrane. Nonspecific binding was blocked by incubation with blocking buffer (5% skim milk in PBS-T), after which the blots were sequentially incubated with primary and secondary antibodies diluted in blocking buffer or primary antibody dilution buffer (20 mM Tris–HCl pH7.5, 150 mM NaCl, 0.1% Tween 20, and 5% BSA; if anti-phospho-RIPK1 was used, PhosSTOP was added) or PBS-T (for secondary antibodies) and analyzed with an ECL Plus Western blotting kit (GE HealthCare, IL, USA). Imaging was performed with LAS-4000 system (Fujifilm, Tokyo, Japan). Densitometry was performed using ImageJ. For reprobing, the PVDF membrane was treated with stripping buffer (62.5 mM Tris–HCl pH6.8, 2% SDS, and 0.7 mL/10 mL of 2-mercaptoethanol) for 30 min at 50 °C. After being washed with PBS-T and incubated with blocking buffer, the membrane was incubated with a suitable primary antibody again.

### qRT-PCR

RNA was purified using RNeasy Mini kit (QIAGEN). After aspiration of the medium, organoid culture were washed once with ice-cold PBS and the lysis buffer was directly added to each well^31^. Using SuperScript IV (Thermo Fisher Scientific), cDNA were synthesized and qPCRs were performed using KAPA SYBR FAST qPCR Master Mix kit (Kapa Biosystems) in an ABI 7500 Real Time PCR System (Thermo Fisher Scientific). The data were analysed with ExpressionSuite Software version 1.3 (Thermo Fisher Scientific). Expression levels were normalized by those of Actb and Gapdh. Primer sequences were obtained from Takara Mouse Housekeeping Gene Primer Set (Actb and Gapdh) and OriGene Tbk1 Mouse qPCR Primer Pair (Tbk1). Sequences of primer pairs were as follows: Actb, catccgtaaagacctctatgccaac, and atggagccaccgatccaca; Gapdh, tgtgtccgtcgtggatctga, and ttgctgttgaagtcgcaggag; Tbk1, gacatgcctctctcctgtagtc, and ggtgaagcacatcactggtctc.

### Statistical analysis

We analyzed data with the two-tailed unpaired *t*-test, one-sample *t*-test, Mann–Whitney *U*-test, one-way ANOVA with Dunnett’s multiple comparison tests, and two-way ANOVA with Bonferroni post-tests, using GraphPad Prism 4 & 8. Differences were considered statistically significant when *P* was less than 0.05.

### Supplementary Information

Supplementary Information.

## Data Availability

The datasets obtained and analysed in the current study are available from the corresponding authors upon appropriate request.
