# Supplementary material for: Housing conditions affect enterocyte death mode and turnover rate in mouse small intestine
Source: Sci Rep. 2023 Nov 22;13:20423. doi: 10.1038/s41598-023-47660-1 (PMC10665386; doi:10.1038/s41598-023-47660-1)
Supplement: Supplementary file 1 — Supplementary Information. [file 41598_2023_47660_MOESM1_ESM.pdf]

Supplementary information

Supplementary Figures: Fig. S1 to Fig. S8

Matsuoka &Tsujimoto Fig. S1

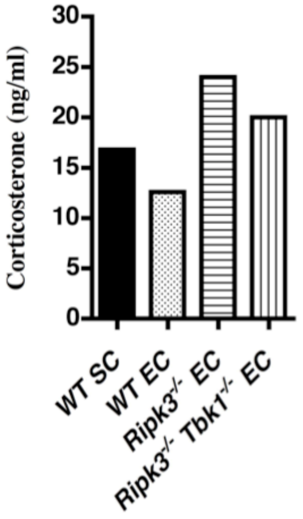

Fig. S1. Plasma corticosterone concentration of mice in various housing conditions

Plasma corticosterone concentrations of a WT mouse housed with Soft Chip (SC) or Eco Chips (EC) and of a *Ripk3*$^{-/-}$ mouse and a *Ripk3*$^{-/-}$ *Tbk1*$^{-/-}$ mouse in Eco Chips (EC) were measured at ZT0 or ZT1 (WT in EC).

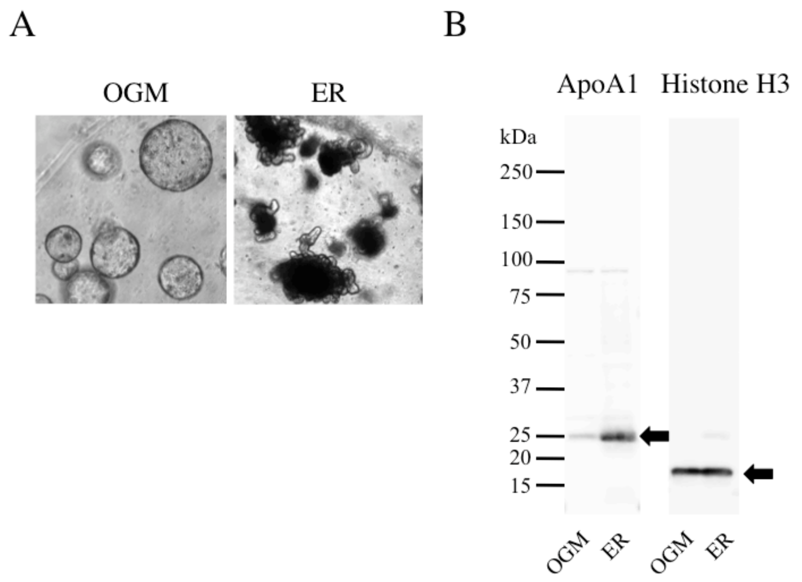

Fig. S2. Small intestinal organoid culture

(A) Duodenal organoids were established as described in Materials and Methods. When they were maintained in organoid growth medium (OGM), they formed spheres. When they were cultured in ER medium (ER), they differentiated and exhibited a darker structure with some buds. We used differentiated organoids in Fig. S3 and Fig. S6. (B) Expression of a differentiation marker, ApoA1 was up-regulated in ER medium. Histone H3 was a loading control. The arrows indicate ApoA1 and Histone H3.

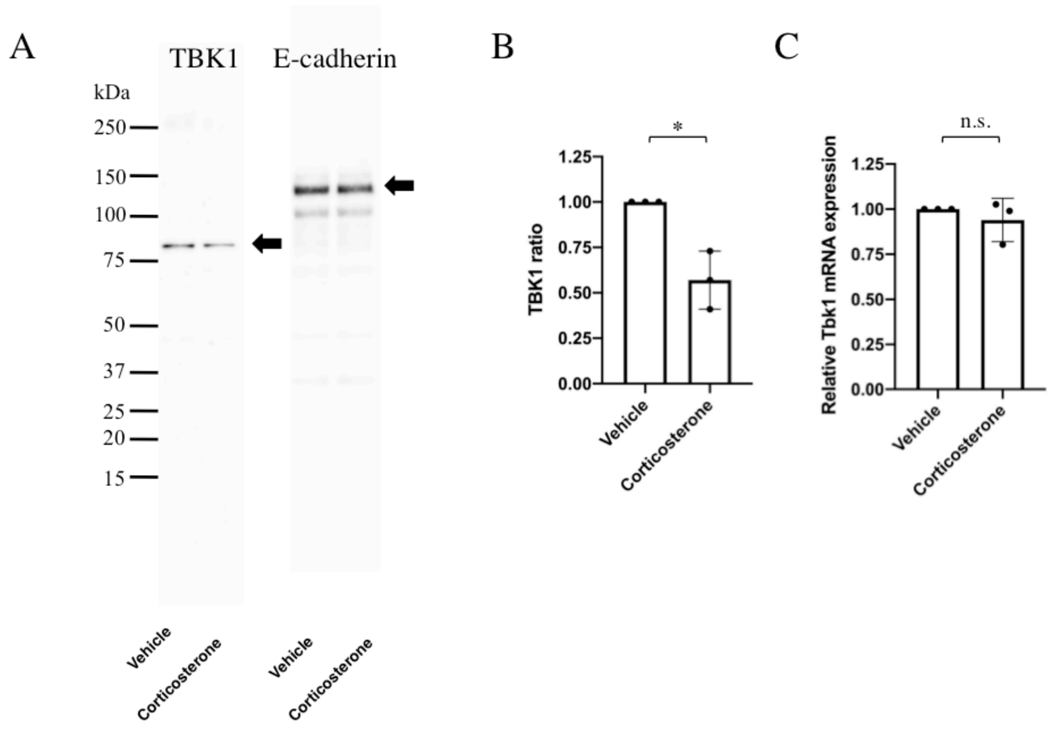

Fig. S3. Effects of corticosterone on TBK1 expression in small intestinal organoids

Duodenal organoids were cultured in ER medium for 3 to 5 days and vehicle or 100 ng/mL corticosterone were added during the last 24 hours. (A, B) The protein amount of TBK1 was decreased in the presence of corticosterone. (A) Western blot analysis of the quantity of TBK1. E-cadherin was a loading control. The arrows indicate TBK1 and E-cadherin. (B) Densitometry of TBK1 was normalized to E-cadherin; then, the ratio (relative to a value of Vehicle as 1) was calculated. The mean ± SEM from three experiments is shown. (C) The expression level of Tbk1 mRNA was not altered significantly in the presence or absence of corticosterone. The expression level of Tbk1 mRNA was examined by real-time RT-PCR analysis. The expression levels were normalized by Gapdh and Actb. The ratio (relative to a value of Vehicle as 1) was

calculated. The mean ± SEM from three experiments is shown. *P* values were calculated using one-sample *t*-tests. *$P<0.05$, n.s., not significant.

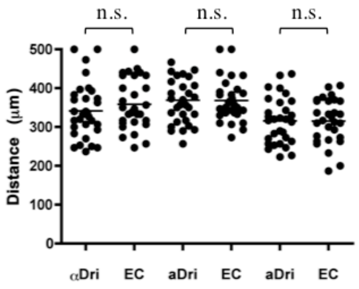

Fig. S4. Enterocyte turnover rates of *Ripk3$^{-/-}$ Tbk1$^{-/-}$*

The distance from the villus-crypt junction to the leading BrdU-labeled cell was measured in each villus of *Ripk3$^{-/-}$ Tbk1$^{-/-}$* mice housed with αDri and compared with the distance in sex-matched littermates housed with Eco Chips (EC) in three independent experiments; the horizontal line represents the mean. *P* values were calculated using two-tailed unpaired *t*-tests. n.s., not significant.

Matsuoka &Tsujimoto Fig. S5

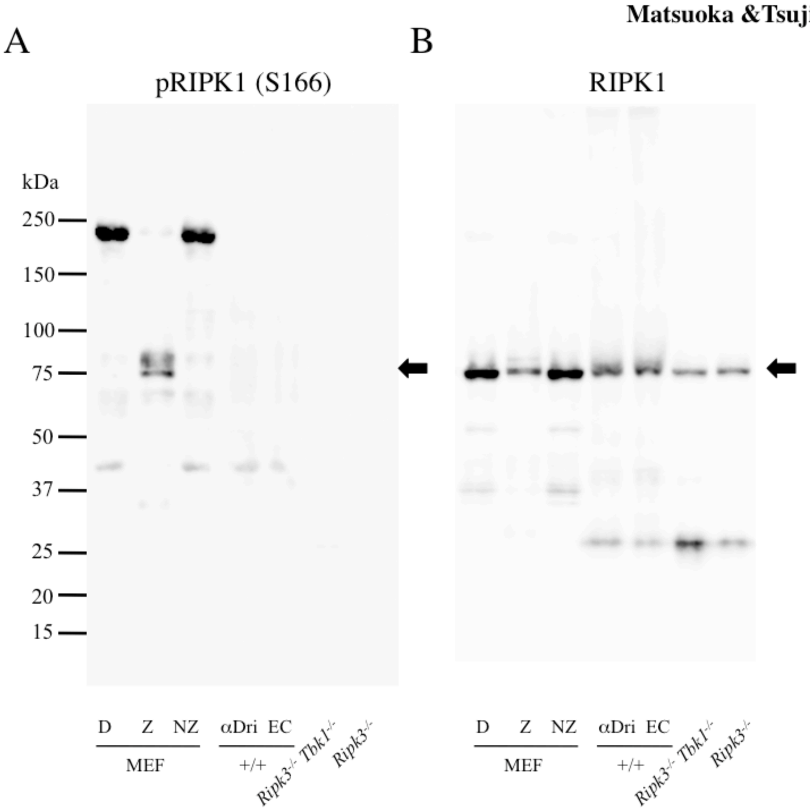

Fig.S5. S166 phosphorylation of RIPK1 was not detected in enterocyte lysates

As a positive control, we prepared lysates from a mouse embryonic fibroblast (MEF) strain which undergoes RIPK1 kinase-dependent cell death with zVAD-fmk as reported in L929 cells[32]. RIPK1 kinase-dependency was confirmed by inhibition of the cell death with necrostatin-1s. We used a popular antibody from Cell Signaling Technology (CST) to detect S166 phosphorylation of RIPK1, however, the background was very high. Therefore, we chose an antibody from Arigo Biolaboratories. (A) The blot using Arigo antibody. The arrow indicates S166 phosphorylated RIPK1. Positive signal was detected in MEF treated with zVAD-fmk (lane Z) but not in MEF treated with zVAD-fmk + Necrostatin-1s (lane NZ), whereas there was no signal in mouse enterocyte lysates. (B) The blot in (A) was reprobed with RIPK1 antibody. The arrow

indicates RIPK1. D: DMSO, Z: 20 μM zVAD-fmk, NZ: 20μM zVAD-fmk + 20μM necrostatin-1s, αDri: a wild type mouse housed with ALPHA-dri, EC: a wild type mouse housed with Eco Chips.

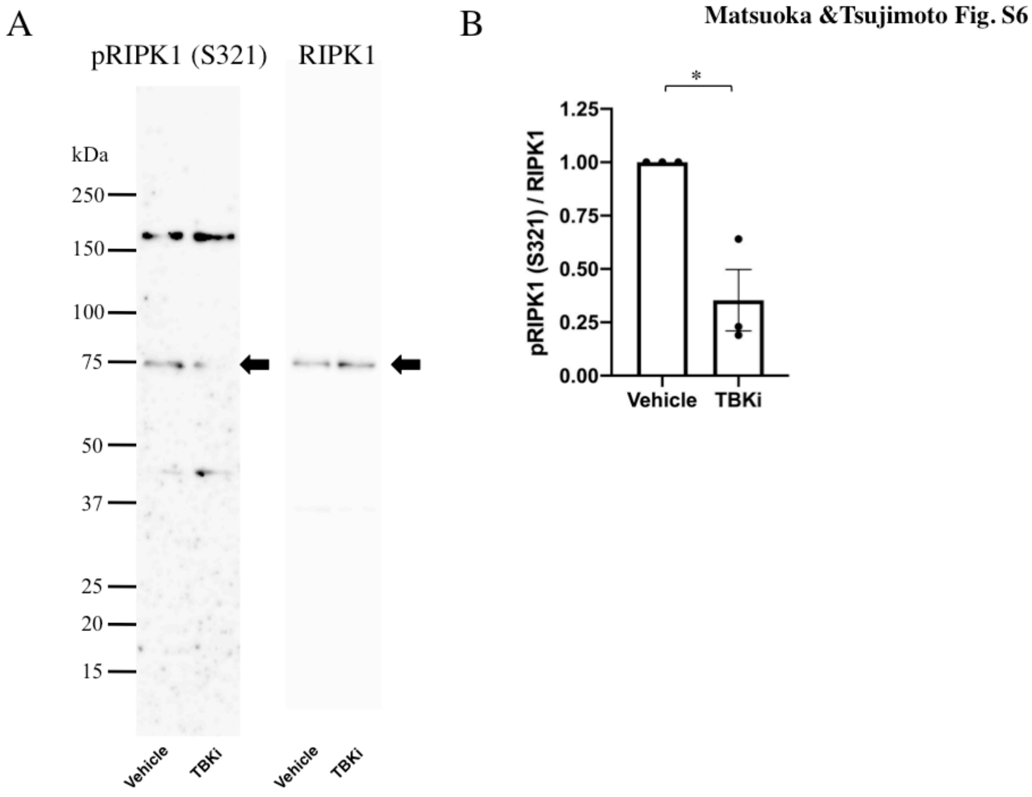

Fig. S6. Effects of TBKi on S321 phosphorylation of RIPK1 in small intestinal organoids

Duodenal organoids were cultured in ER medium for 3 to 5 days and then vehicle or 2 μM TBKi were added for 2 hours. (A, B) The level of S321-phosphorylated RIPK1 was decreased in the presence of TBKi. (A) Western blots showing the level of S321-phosphorylated-RIPK1 and total RIPK1. The arrows indicate S321 phosphorylated RIPK1 and total RIPK1. (B) Densitometry was performed to obtain the ratio of p-RIPK1 (S321) to total RIPK1. The mean ± SEM from three experiments is shown. *P* values were calculated using one-sample *t*-tests. **P*<0.05.

Matsuoka &Tsujimoto Fig. S7

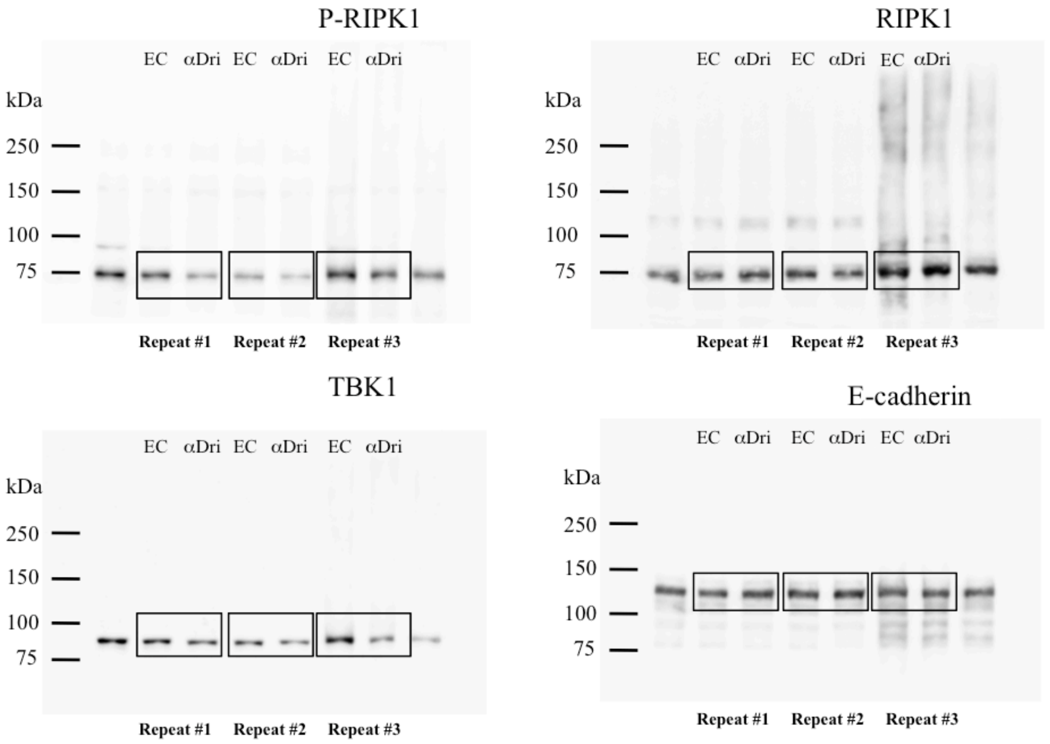

Fig. S7. Uncropped Western blots for Figure 4G, H, and I

All samples were run on a single gel and subjected to Western blot analysis for P-RIPK1. The blot was reprobed for three different proteins as indicated. The samples from three independent experiments are represented by Repeat #1, 2, and 3. Repeat #1, 2, and 3 were used for quantification in Figures 4H and 4I. Repeat #3 was shown as representative data in Figure 4G.

Matsuoka &Tsujimoto Fig. S8

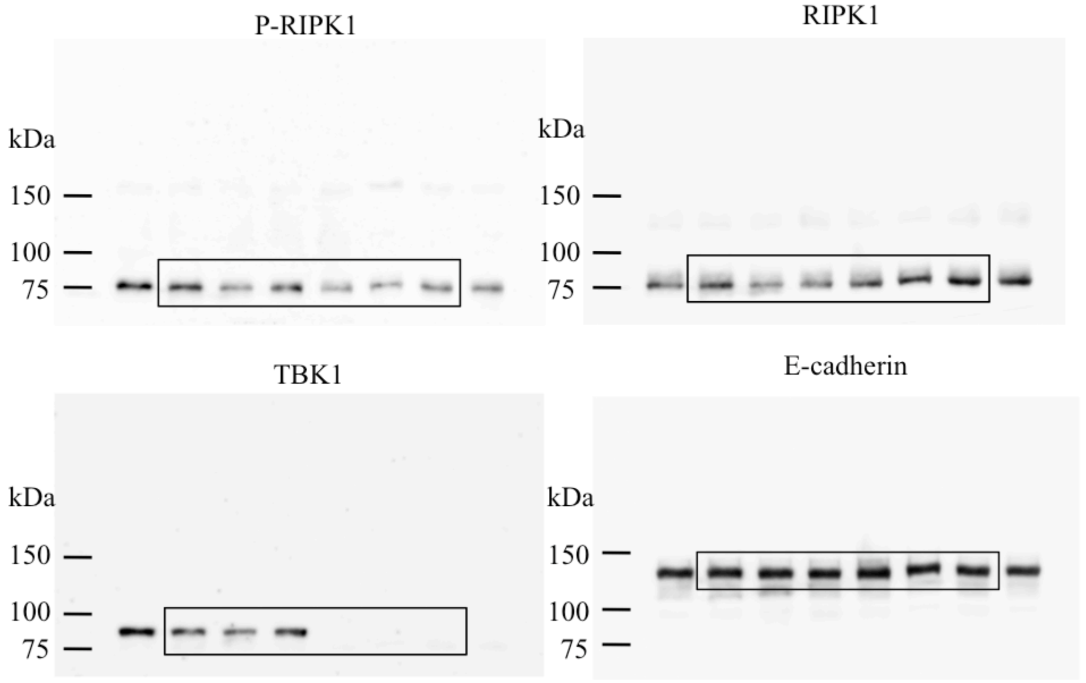

Fig. S8. Uncropped Western blots for Figure 5D

All samples were run on a single gel and subjected to Western blot analysis for P-RIPK1. The blot was reprobed for three different proteins as indicated.
